# A Nonsense Mutation in COL4A4 Gene Causing Isolated Hematuria in Either Heterozygous or Homozygous State

**DOI:** 10.3389/fgene.2019.00628

**Published:** 2019-07-02

**Authors:** Cheng Yang, Yuan Song, Zhaowei Chen, Xiaohan Yuan, Xinhua Chen, Guohua Ding, Yang Guan, Mary McGrath, Chunhua Song, Yongqing Tong, Huiming Wang

**Affiliations:** ^1^Renal Department, Renmin Hospital of Wuhan University, Wuhan, China; ^2^Ultrastructure Center, Renmin Hospital of Wuhan University, Wuhan, China; ^3^Department of Pediatrics, Pennsylvania State University College of Medicine, Hershey, PA, United States; ^4^Department of Laboratory Science, Renmin Hospital of Wuhan University, Wuhan, China

**Keywords:** human genetics, collagen type IV, Alport syndrome, *COL4A4*, genomic variant, hereditary nephropathy

## Abstract

Alport syndrome (AS) is a hereditary nephropathy characterized by glomerular basement membrane lesions. AS shows a relatively rare entity with autosomal dominant gene mutation (accounts for less than 5% of AS cases) and is widely believed to be a consequence of heterozygous variants in the ***COL4A3*** and ***COL4A4*** genes. Until now, there have been no reports of homozygous variants in genes in AS patients, and it is scarce to detect both homozygous and heterozygous variants in a single AS pedigree. We performed genetic analysis by exome sequencing (exome-seq) in a Chinese family with AS and found four individuals harboring the ***COL4A4*** c.4599T > G variant, a novel *COL4A4* nonsense mutation that gains stop codon and results in a truncated protein. The proband and her two siblings were determined to be heterozygous, whereas their mother was homozygous. The proband satisfied the criteria for the diagnosis of AS, which included clinical manifestations of microscopic hematuria and proteinuria, and pathological features of the glomerular basement membrane (GBM), including irregular thickening and splitting. However, the other three individuals who were homozygous or heterozygous for the variant exhibited mild clinical features with isolated microscopic hematuria. In summary, we identified a novel pathogenic variant in either the heterozygous or homozygous state of the ***COL4A4*** gene in a Chinese family with AS. Our results also suggest that the severity of clinical manifestations may not be entirely attributed to by the ***COL4A4*** genetic variant itself in patients.

## Introduction

Alport syndrome (AS) is a genetic disorder characterized by glomerulonephritis, end-stage kidney disease, ocular abnormalities, and hearing loss. It is caused by pathogenic variants in the *COL4A3*, *COL4A4*, and *COL4A5* genes, which encode the α3, α4, and α5 chains of collagen type IV (COL4) respectively. The glomerular basement membrane (GBM) contains three layers, including the lamina lucida interna, lamina densa, and lamina lucida externa. COL4 is a major component of the GBM of the kidney. Pathogenic variants involving this gene result in the splitting of the GBM or more severe pathological lesions involving the kidney (Kruegel et al., 2013). More than 1,700 unique variants have been identified in these genes and involve three inheritance patterns: X-linked AS (XLAS), autosomal recessive AS (ARAS), or autosomal dominant AS (ADAS). XLAS is associated with variants in the *COL4A5* gene and accounts for 85% of patients with AS, while ARAS is found with variants in the *COL4A3* and *COL4A4* genes and accounts for 15% of AS patients (Hudson et al., 2003; Nagel et al., 2005; Hertz et al., 2015), and ADAS is seen with variants in the *COL4A3* and *COL4A4* genes, accounting for 5% of AS patients (Lemmink et al., 1996).

The correlation of genotype and phenotype between COL4 gene mutations and the clinical manifestations of AS have been previously described. XLAS is a highly penetrant disease in hemizygous males, and 70% of these cases rapidly progress to end-stage renal disease (ESRD) before the age of 30. Only a few cases (30%) reach ESRD after 30 years of age (Flinter et al., 1988; Myers et al., 1990; Jais et al., 2000). However, in heterozygous female patients with XLAS, clinical features are variable, ranging from isolated micro-hematuria to ESRD (Rheault, 2012). ARAS, which is a consequence of homozygous variants or compound heterozygous variants in the *COL4A3* and *COL4A4* genes, usually involves severe early disease in both females and males (Longo et al., 2006; Oka et al., 2014). ADAS has been ascribed to heterozygous variants in the *COL4A3* or *COL4A4* gene, and clinical manifestations usually include mild and slowly progressing renal disease compared with that in patients with ARAS or males with XLAS(7). Theoretically, the asymptomatic, late-onset, or mildly affected individuals with the autosomal dominant disease may not be diagnosed; consequently, the offspring of two carrier parents may have possibilities to carry two variant alleles and present with a homozygous state. In reality, homozygotes have not yet been described for AS, although they are reported in some pedigrees of autosomal dominant diseases (Chemke et al., 1984; Brandi et al., 1993; Tonini et al., 2004; Arzel-Hezode et al., 2010; Vinciguerra et al., 2014; Conidi et al., 2015; Kosik et al., 2015; Sjouke et al., 2015). In this study, we identified a novel mutation, c.4599T > G (p.Tyr1533Ter), in the *COL4A4* gene, which is associated with AS disorder in three generations of a Chinese family. Of particular interest is the coexistence of homozygote and heterozygote carriers in a single pedigree, which, to our knowledge, has not been reported in the literature.

## Results

### Clinical Characteristics of the Pedigree

Seven members of this family, including four males and three females, did not have high blood pressure, ophthalmic impairment, or audiological abnormalities. The proband (II:3) manifested proteinuria (2.3 g per day), micro-hematuria, and a mildly elevated serum creatinine level (92.5 µmol/L). Her mother and two of her siblings (one brother and one sister) displayed isolated micro-hematuria and normal serum creatinine levels. Her father, her husband, and her son showed no symptomatic glomerulopathy. Consanguinity was denied by the family members. The clinical characteristics of the family are summarized in **Table 1**.

**Table 1:** Clinical and genetic data of the family with Alport syndrome (AS).

Subject	I:1	I:2	II:1	II:2	II:3	III:1
Sex	M	F	F	M	F	M
Age (years)	83	80	59	52	46	23
Blood pressure	Normal	Normal	Normal	Normal	Normal	Normal
Hematuria	No	3+	2+	2+	3+	No
Proteinuria	No	No	No	No	3+	No
BUN (mmol/L)	6	7.5	Normal	Normal	6.13	Normal
Scr (µmol/L)	81.5	91.3	Normal	Normal	92.5	Normal
Audiological examination	Normal	Normal	Normal	Normal	Normal	Normal
Ophthalmic examination	Normal	Normal	Normal	Normal	Normal	Normal
Genotype	−	Hom	Het	Het	Het	−

BUN, blood urea nitrogen; Scr, serum creatinine values; Hom, homozygous; Het, heterozygous.

## Renal Pathology Manifestation

**Figure 1:**
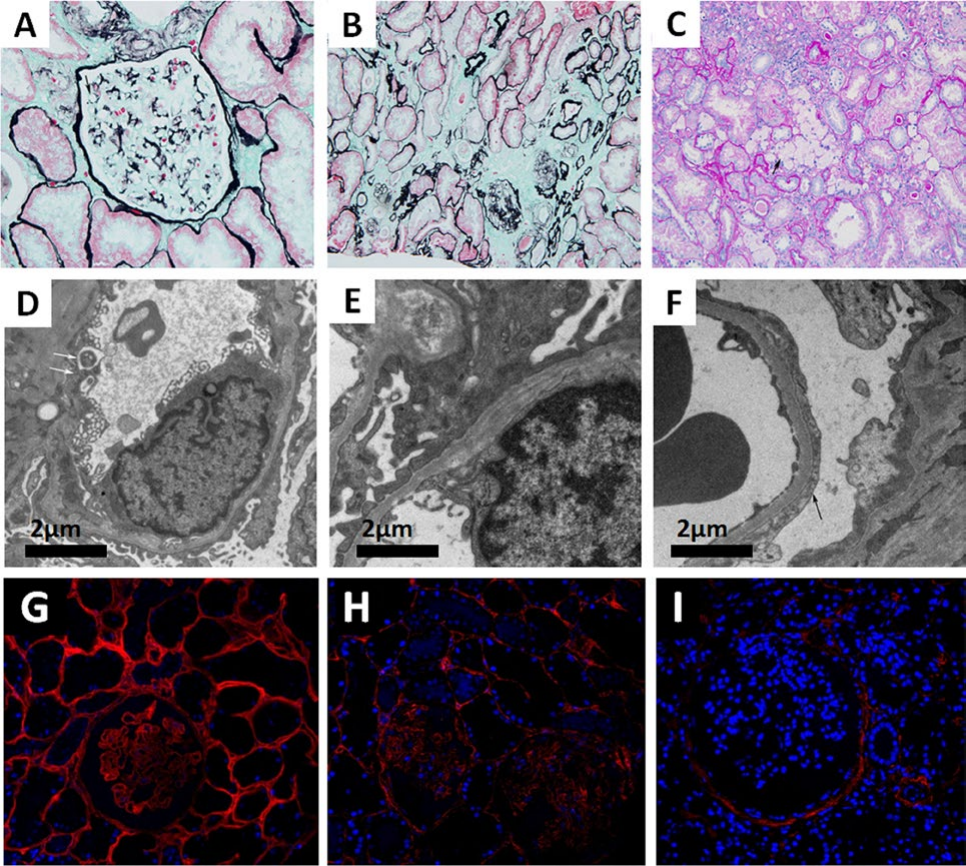
**(A–C)** Images of renal pathology (II:3) under light microscopy by periodic acid-silver methe-namine (PASM) **(A** and **B)** and periodic acid-schiff (PAS) staining **(C)**. A non-uniform glomerular basement membrane (GBM) (arrow), 200×. **(B)** Tubular atrophy and interstitial fibrosis, 200×. **(C)** The infiltration of foam cells (arrow) in renal interstitium. **(D–F)** Representative electron micrograph of renal tissue. **(D)** Some segments of GBM were absent (arrow). **(E)** Irregular GBM thinning, thickening, and splitting. **(F)** Effacement of podocyte foot processes (arrow). **(G–I)** Immunofluorescence staining of Col4A3 (Ab111742, Abcam, USA) in renal tissue from a patient with minimal change disease (MCD) (**G**, left panel), the proband (II:3) (**H**, middle panel), and a male patient with XLAS (**I**, right panel), 400×.

Seventeen glomeruli were available and assessed under a light microscope. Glomerular sclerosis was observed in two of the glomeruli. Among the remaining 15 glomeruli, the glomerular capillary tuft was normal by light microscopy. The tubules showed dispersed granular degeneration and cast formation. The tubular casts included blood cell casts and proteins casts. The areas of the brush border in the kidney tubules were significantly smaller. Interstitial inflammatory cell infiltration and foam cells were observed focally. The intima of the arterioles showed thickening with occasional hyaline changes (**Figure 1A–C**). Immunofluorescence staining showed granular IgM deposition in the mesangium, whereas staining for IgG, IgA, C3, C1q, and C4 was all negative. The ultramicroscopic evaluation revealed that most segments of the GBM were normal, whereas some exhibited irregular thinning (<200 nm) and thickening or splitting. There was localized effacement of podocyte foot processes. No distinct dense deposits in the glomerular basement membrane or in the mesangium were detected (**Figure 1D–F**). For Col4 expression profiling, Col4 immunofluorescence staining was performed on the renal tissues of a patient with minimal change disease (MCD), the proband (II:3), and a male patient with XLAS. The results indicated that although Col4 was positively expressed in the glomeruli of the proband (II:3), it was weaker than that of the MCD patient. As a negative control, no Col4 was observed in the glomeruli of the male patient with XLAS (**Figure 1G–I**).

## Genomic DNA Extraction and Exome Sequencing

The total DNA concentration of the sample from the proband (II:3) was 68.2 ng/μl, and the ratio of the absorbance at wavelengths of 260 and 280 nm (A260/280) was 1.83, indicating that the isolated DNA was pure. Exome sequencing was also performed to identify sequence variants. A total of 293,903 amplicons were generated by the polymerase chain reaction (PCR) method. Approximately 57,742,646 base pairs were identified in the targeted regions. The average base coverage depth was 114.6×. The uniformity of coverage was 93.24%. These results are summarized in **Table 2**.

**Table 2:** Comparison of secondary structure between wild-type COL4A4 and the mutant protein.

	Wild type		Mutant	
	Number	Percentage (%)	Number	Percentage (%)
Alpha helix	58	3.43	15	0.98
310 helix	0	0	0	0
Pi helix	0	0	0	0
Beta bridge	0	0	0	0
Extended strand	185	10.95	142	9.27
Beta turn	138	8.17	124	8.09
Bend region	0	0	0	0
Random coil	1,309	77.46	1,251	81.66
Ambiguous states	0	0	0	0

## Variant Analysis and COL4A4 Variant Screening

**Figure 2:**
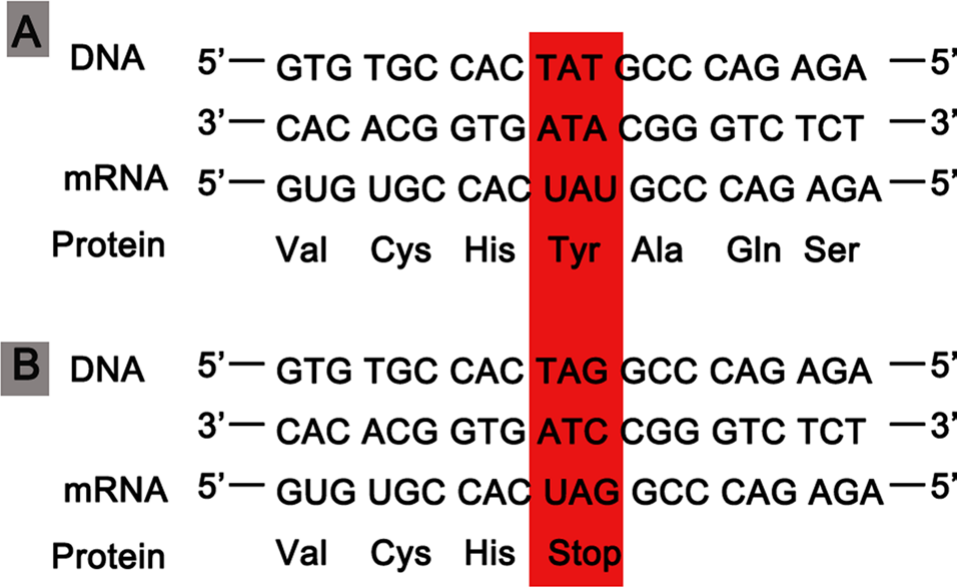
**(A)** The process of gene expression in the normal wild-type COL4A4. **(B)** A variant codon in the DNA sequence (TAG) leading to a nonsense change from tyrosine to a stop codon (UGA).

**Figure 3:**
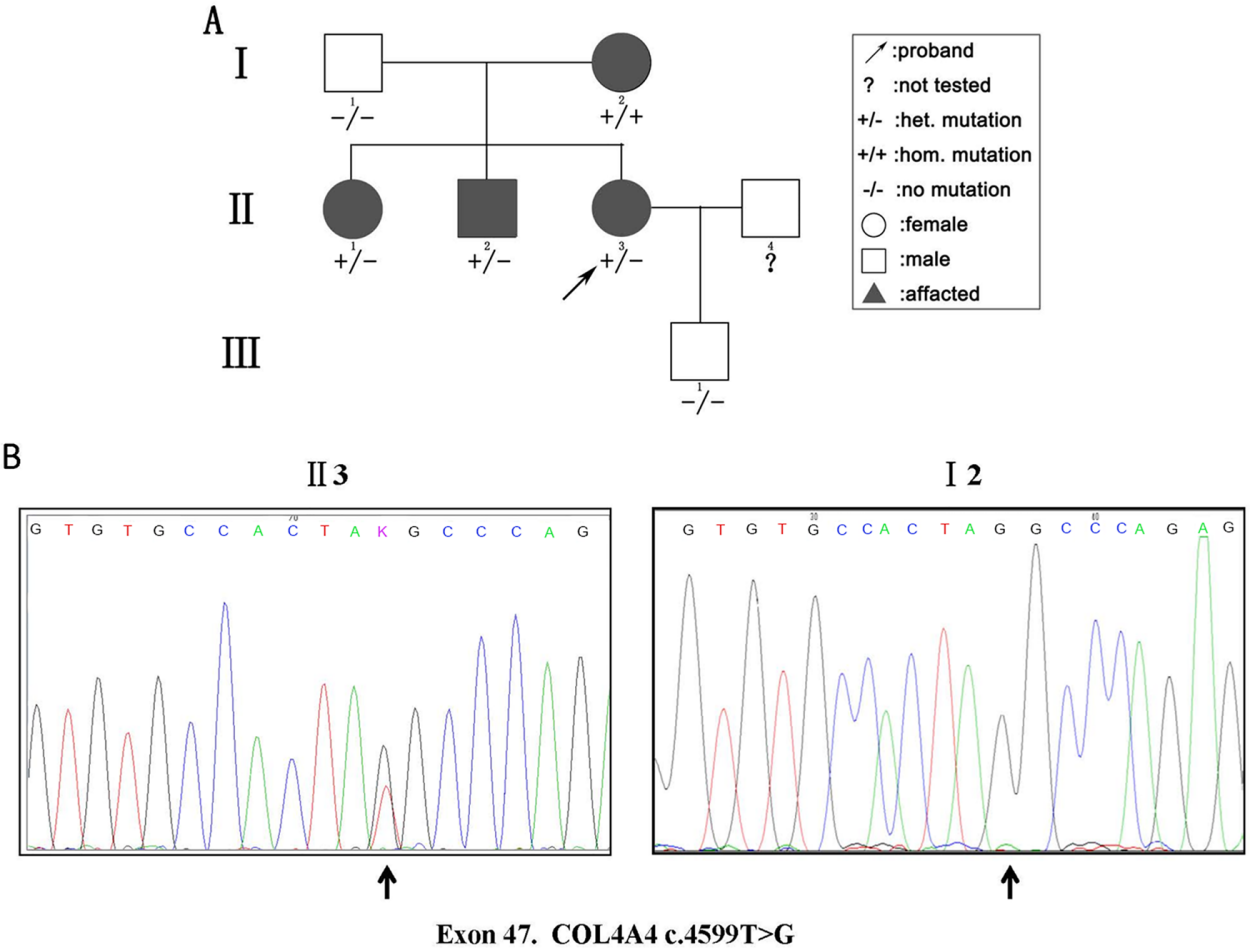
**(A)** Pedigree of a three-generation family with AS. Arrow indicates the proband. The proband, her brother, and her sister were heterozygous (het). For the novel pathogenic variant, whereas the mother was homozygous (hom). The shapes filled with black indicate patients affected with hematuria. **(B)** Sequencing results of the c.4599T > G (p.Tyr1533Ter) variant at the 47th exon of the COL4A4 gene of the proband (II:3) (left panel) and her mother (I:2) (right panel).

We detected 583 variants in the proband (II:3) and filtered out synonymous and intronic variants using public databases. Variants in genes related to proteinuria were all analyzed in terms of pathogenicity. A novel heterozygous variant c.4599T > G in exon 47 of the *COL4A4* gene was classified as pathogenic. The c.4599T > G variant was predicted to result in a premature translation termination codon (TAT > TAG) at amino acid position 1,533 (p.Tyr1533Ter), and the remaining codons of the mRNA were not translated into amino proteins (**Figure 2**). The variant was confirmed by Sanger sequencing and her family members (I:1, I:2, II:1, II:2, and III:1; **Figure 3A**) were subsequently assessed. The c.4599T > G (p.Tyr1533Ter) *COL4A4* variant was not detected in her father or her son, whereas her mother was homozygous (**Figure 3B**), and her sister and brother were heterozygous. The results are shown in **Table 1**.

## Bioinformatics Analysis of the Novel *COL4A4* Variant

The tyrosine at position 1,533 (p.Tyr1533) is conserved across primates from humans (*Homo sapiens*) to *Nomascus leucogenys* (data not shown). Such a scenario indicates that a sequence variant at this position may negatively affect protein structure and function. Secondary structure analysis predicted that the wild-type COL4A4 contains 58 alpha helices and 1,309 random coils, whereas the variant sequence only consists of 15 alpha helices and 1,251 random coils (**Table 2**).

## Discussion

Collagen IV is the main component of the GBM lamina densa (Laurie et al., 1984) and is encoded by six genes (COL4A1–COL4A6) (Harvey et al., 2006). Variants in the *COL4A3/A4/A5* genes result in type IV collagen nephropathies, which include AS, thin basement membrane nephropathy (TBMN) (Pierides et al., 2013; Gross et al., 2017; Imafuku et al., 2017; Daga et al., 2019; Lee et al., 2019), and focal segmental glomerulosclerosis (FSGS) (Voskarides et al., 2008; Wu et al., 2016). Here, we report of a Chinese family with AS that involves a novel variant, COL4A4 4599T > G, which occurred in either the homozygous or heterozygous state in affected individuals with mild kidney involvement.

Previous studies have described a causal relationship between heterozygous *COL4A3/COL4A4* variants and FSGS (Voskarides et al., 2007; Pierides et al., 2009). Due to this finding, some patients with ADAS may have been diagnosed as familial FSGS (Malone et al., 2014; Xie et al., 2014; Gast et al., 2016). FSGS is one of the major causes of renal failure and ESRD (Korbet, 2012). Large proteinuria (>3 g per day) with hypertension, microscopic hematuria, and kidney failure are common in patients with FSGS. Segmental sclerosis and podocyte lesions are the prominent histological changes in FSGS. These features, either clinical or pathological, were not detected in this family, thus leading to the exclusion of a diagnosis of familiar FSGS.

Inherited glomerular hematuria affects at least 1% of the population and is mainly caused by TBMN and less often is due to AS (Savige et al., 2003; Tryggvason and Patrakka, 2006). Unlike the clinical feature of TBMN, which usually manifests with isolated hematuria, AS is typically characterized by hematuria, proteinuria, renal function impairment, and sensorineural deafness and ocular abnormalities. Besides the difference in clinical appearances, TBMN and AS also have different genetic natures. As a hereditary disease, AS is caused by pathogenic variants in the *COL4A3*, *COL4A4*, and *COL4A5* genes. COL4A5 pathogenic variants usually cause **X**-linked AS, whereas those involving the *COL4A3* and *COL4A4* genes generally lead to autosomal dominant or autosomal recessive types (Lemmink et al., 1996; Hudson et al., 2003; Nagel et al., 2005; Hertz et al., 2015). Hereditary TBMN is a disorder caused by pathogenic variants in the *COL4A3* and *COL4A4* genes, often presenting as an autosomal dominant genetic disease (Savige et al., 2003), although some biopsy-proven TBMN individuals were found to be carriers of autosomal recessive AS (Lemmink et al., 1996; Boye et al., 1998; Netzer et al., 1999; Buzza et al., 2001). In this report, we describe a three-generational family with autosomal dominant AS and not autosomal TBMN because the proband in this family clinically manifested with proteinuria and hematuria. In addition, the renal pathological changes in the proband displayed GBM irregular thinning and thickening or splitting but no uniform thinning.

ADAS is generally a mild and slowly progressing disease. Its clinical manifestations, especially in younger patients, seem to be significantly milder than those in men with XLAS or patients with ARAS (Jais et al., 2000; AegeanSoftware. NoteExpress, 2005), and its extra-renal manifestations are relatively rare (Jais et al., 2000; Oka et al., 2014). No identifiable phenotype–genotype correlation has been observed in patients with ADAS because their phenotype varies regardless of genetic background. In the pedigree described in this study, most of the individuals carrying the novel pathogenic variant exhibited isolated hematuria without the extra-renal phenotype, except for the proband who presented with hematuria and proteinuria. Particularly, an 80-year old woman (I:2) carrying the homozygous COL4A4 pathogenic mutation also only has the isolated hematuria. This indicates that the COL4A4 c.4599T > G variant does lead to hematuria, but by itself, it is not enough to cause AS, whether in homozygous or heterozygous individuals. Previous studies have shown that functional haplo-insufficiency for *COL4A3* or *COL4A4* results in hematuria but does not always lead to AS (Lemmink et al., 1997; Boye et al., 1998). There are two possible explanations for this complex ADAS phenotype. First, the nature of the variant causes defects in the position and function of amino acids, thereby resulting in phenotypic manifestations or penetration. With respect to the pedigree, the c.4599T > G variant, which is a nonsense mutation and located in the NC1 domain of the *COL4A4* gene, leads to a truncated NC1 α chain. We presume that this shortened α chain may not influence the assembly of the collagen IV protein but just affects the integrity of the collagen IV network, thus not resulting in a severe renal phenotype. Second, except for the alteration in the structure and function of collagen IV that is caused by the mutation itself, the complex phenotype of ADAS could also be attributed to other factors, such as environmental or genetic factors. For example, hypertension and obesity have been identified as contributory factors, and several genetic modifiers were also reported as epistatic factors that regulate the phenotype (Pereira et al., 2004; Kottgen et al., 2008; Deltas et al., 2013; Pierides et al., 2013; Stefanou et al., 2015; Kamiyoshi et al., 2016; Gross et al., 2017; Daga et al., 2019; Lee et al., 2019).

In this family, we found that the proband, who was heterozygous for the pathogenic variant, exhibited more severe renal symptoms than her mother, who was homozygous for this variant. Autosomal dominant hereditary diseases are generally exhibited in heterozygous individuals carrying a pathogenic variant; homozygous individuals are rarely observed and often result from assortative mating or consanguinity. A few cases of homozygotes in the disease pedigree have been previously reported (Chemke et al., 1984; Brandi et al., 1993; Zlotogora, 1997; Tonini et al., 2004; Arzel-Hezode et al., 2010; Vinciguerra et al., 2014; Conidi et al., 2015; Kosik et al., 2015; Sjouke et al., 2015), and their clinical phenotype is usually more severe than that of the heterozygous individuals (Chemke et al., 1984; Arzel-Hezode et al., 2010; Vinciguerra et al., 2014; Conidi et al., 2015; Kosik et al., 2015). However, exceptions to homozygotes displaying milder phenotypes have also been reported (Zlotogora, 1997; Tonini et al., 2004; Sjouke et al., 2015), and these individuals not only develop clinical symptoms at a later age but also exhibit slower disease progression than do heterozygotes. An important reason seen, as mentioned above, is that in addition to the alteration in the structure and function of the protein caused by the mutation itself, other factors, such as environmental or genetic factors, also play important roles in the manifestation severity of ADAS (Pereira et al., 2004; Kottgen et al., 2008; Deltas et al., 2013; Stefanou et al., 2015; Kamiyoshi et al., 2016).

To our knowledge, homozygous AS has not been reported in the literature. In this AS pedigree, one homozygote was found and manifested with a milder renal phenotype than the heterozygous proband. This is similar to previously reported cases of other diseases and indicates that the phenotype of kidney disease in patients with AS is determined by multiple factors in addition to the genetic background of a single gene. We will continue to follow up the family and do whole genome sequencing to further identify other possible reasons in the future. The molecular mechanism underlying the variations in phenotypic consequences remains elusive and thus needs further investigation using cellular and/or animal models.

In summary, ADAS has long been considered as a consequence of heterozygous pathogenic variants in the *COL4A3* and *COL4A4* genes, and homozygous carriers have never been reported in an ADAS family. We have described one Chinese pedigree with ADAS that involves a novel pathogenic variant, COL4A4 c.4599T > G, which is predicted to result in ADAS, yet its pedigree includes both homozygous and heterozygous carriers. Our results show that the proband with the heterozygous variant state had severe clinical manifestations than had others with either homozygous or heterozygous variant. This suggests that the severity of clinical manifestations may be attributed to not only the genetic variants but also other unknown factors.

## Materials and Methods

### Family History

This study has been approved by the Ethics Committee of Renmin Hospital of Wuhan University (Wuhan City, Hubei Province of China), and written informed consent was obtained from the parents or guardians of the participant for the publication of this case report. Three generations of a Chinese family consisting of seven members were included in this research. Urinalysis and renal function evaluation were performed on all included family members. Peripheral blood samples were also obtained for next-generation sequencing, including four affected members (I:2, II:1, II:2, and II:3; **Figure 3A**) and two unaffected individuals (I:1 and III:1; **Figure 3A**). The whole family also underwent routine examination for renal function, vision, and hearing. Renal biopsy was performed on the proband of the family (II:3).

### Renal Biopsy and Renal Pathology Examination

A renal biopsy of the proband (II:3) was collected on the condition that this procedure had no contraindications. With the assistance of sonography, a nephrologist inserted a biopsy needle into the kidney under local anesthesia and successfully obtained a tissue sample. The pathologists then evaluated the tissue by light, electron, and fluorescence microscopy.

### Genomic DNA Extraction (gDNA) and Exome Sequencing

gDNA was isolated from peripheral blood of six members (I:1, I:2, II:1, II:2, II:3, and III:1). Exome sequencing of gDNA was performed using the Life Technologies Proton sequencer by the laboratory medicine department of Renmin Hospital, Wuhan, China. A DNA library was constructed using the Ion AmpliSeq^™^ Exome Enrichment Kit (Life Technologies, USA), and templates were prepared using Ion PGM^™^ Enrichment Beads (Life Technologies, USA) according to the manufacturer’s recommendation. Then, sequencing was performed using an Ion PI^™^ Chip Kit v2 (Life Technologies, USA), and data analysis was performed by comparing the results to the sequences of the AS-associated genes (COL4A3–COL4A6) using the Ion Reporter^™^ software (Life Technologies, USA).

### Variant Analysis

All synonymous and intronic variants were filtered out using the data from public databases, including the ClinVar database (https://www.ncbi.nlm.nih.gov/clinvar/), the UCSC database (https://genome.ucsc.edu/), the single nucleotide polymorphism database (dbSNP, http://www.ncbi.nlm.nih.gov/projects/SNP/snp_summary.cgi), and 1000 Genomes Project (http://www.1000genomes.org/). After the benign variants were excluded, the remaining variants were designated as novel. We performed Sanger sequencing for variant confirmation and searched the HGMD database (http://www.hgmd.cf.ac.uk/ac/index.php) and PubMed database (http://www.ncbi.nlm.gov/pubmed) to predict the potential gene dysfunction.

### Bioinformatics Analysis of the Detected Sequence Variants

We compared the nucleotide sequences with sequence databases, and we aligned multiple sequences among various species using the Basic Local Alignment Search Tool (https://blast.ncbi.nlm.nih.gov/Blast.cgi). In addition, we also searched for protein sequences and functional information in UniProt databases (http://www.uniprot.org). For secondary structure comparison between the wild-type and variant proteins, we used the single nucleotide polymorphism (NPS) server (https://prabi.ibcp.fr/htm/site/web/home). The identified new nonsense mutant, *COL4A4* c.4599T > G (p.Tyr1533Ter), was submitted to ClinVar database with a submission ID SUB5664647.

## Contribution to the Field

We performed the genetic analyses by exome-seq in a Chinese family with AS and found four individuals harboring the ***COL4A4*** c.4599T > G variant in the family. We identified a novel pathogenic variant in either the heterozygous or homozygous state of the ***COL4A4*** gene in this family. Our results also suggest that the severity of clinical manifestations may not be entirely attributed to the ***COL4A4*** genetic variant itself in patients.

## Consent for Publication

All participants signed a consent form for publication.

## Data Availability Statement

The datasets supporting the conclusions of this article are available in ClinVar (http://www.ncbi.nlm.nih.gov/clinvar/).

## Ethics Statement

This study has been approved by the Ethical Committee of Renmin Hospital of Wuhan University, and written informed consent was obtained from the parents or guardians of the participant for the publication of this case report.

## Author Contributions

HW: Manuscript writing and overall instruction.

CY: Manuscript writing and data collection.

YS: Manuscript writing and data collection.

ZC: Fluorescence microscopic examination.

XY: Pathology materials preparation.

XC: Clinical data collection.

GD: Clinical data collection.

YG: Electron microscope examination.

YT: Sequencing.

CS: Manuscript writing and preparation.

MM: Manuscript writing and preparation.

## Funding

The National Natural Science Foundation of China (81370800) and National Institute of Hospital Administration (an Explorer Program to Promote the Application of Peritoneal Dialysis in China) supported this study.

## Conflict of Interest Statement

The authors declare that the research was conducted in the absence of any commercial or financial relationships that could be construed as a potential conflict of interest. The results presented in this paper have not been published previously in whole or in part, except for an abstract.
